# The cAMP-dependent phosphorylation footprint in response to heat stress

**DOI:** 10.1007/s00299-024-03213-y

**Published:** 2024-05-07

**Authors:** Guido Domingo, Milena Marsoni, Eleonora Davide, Stefania Fortunato, Maria Concetta de Pinto, Marcella Bracale, Gianluca Molla, Chris Gehring, Candida Vannini

**Affiliations:** 1 Biotechnology and Life Science Department, University of Insubria, Via Dunant 3, 21100 Varese, Italy; 2 Department of Biology, University of Bari “Aldo Moro”, Piazza Umberto I, 70121 Bari, Italy; 3 Department of Chemistry, Biology and Biotechnology, University of Perugia, Borgo XX Giugno, 74, 06121 Perugia, Italy

**Keywords:** Protein phosphorylation, Kinases, Cyclic adenosine monophosphate, cAMP, Heat stress, Systemic response, RNA processing

## Abstract

**Key message:**

cAMP modulates the phosphorylation status of highly conserved phosphosites in RNA-binding proteins crucial for mRNA metabolism and reprogramming in response to heat stress.

**Abstract:**

In plants, 3′,5′-cyclic adenosine monophosphate (3′,5′-cAMP) is a second messenger that modulates multiple cellular targets, thereby participating in plant developmental and adaptive processes. Although its role in ameliorating heat-related damage has been demonstrated, mechanisms that govern cAMP-dependent responses to heat have remained elusive. Here we analyze the role cAMP–dependent phosphorylation during prolonged heat stress (HS) with a view to gain insight into processes that govern plant responses to HS. To do so, we performed quantitative phosphoproteomic analyses in *Nicotiana tabacum* Bright Yellow-2 cells grown at 27 °C or 35 °C for 3 days overexpressing a molecular “sponge” that reduces free intracellular cAMP levels. Our phosphorylation data and analyses reveal that the presence of cAMP is an essential factor that governs specific protein phosphorylation events that occur during prolonged HS in BY-2 cells. Notably, cAMP modulates HS-dependent phosphorylation of proteins that functions in mRNA processing, transcriptional control, vesicular trafficking, and cell cycle regulation and this is indicative for a systemic role of the messenger. In particular, changes of cAMP levels affect the phosphorylation status of highly conserved phosphosites in 19 RNA-binding proteins that are crucial during the reprogramming of the mRNA metabolism in response to HS. Furthermore, phosphorylation site motifs and molecular docking suggest that some proteins, including kinases and phosphatases, are conceivably able to directly interact with cAMP thus further supporting a regulatory role of cAMP in plant HS responses.

**Supplementary Information:**

The online version contains supplementary material available at 10.1007/s00299-024-03213-y.

## Introduction

Plants are highly sensitive to heat stress (HS) during all stages of their growth and development. High temperatures lead to the production of toxic levels of reactive oxygen species (ROS), protein misfolding and denaturation, protein synthesis inhibition, and reduced RNA stability, among others, thereby disrupting cellular homeostasis and altering fundamental physiological processes such as photosynthesis, transpiration, respiration, nutrient uptake, and transport, which all contribute to crop yield and quality (Iizumi et al. 2017).

The sessile nature of plants necessitated the evolution of an intricate network of molecular mechanisms that contribute to various degrees of adaptive acclimation to high-temperature conditions (Bigot et al. 2018; Haider et al. 2021; Saini et al. 2022; Zhao et al. 2021). Deciphering these processes is challenging since they are complex, multi-leveled, show species-specific variations, and are dependent on the duration, increment, and frequency of temperature increases.

In plants, like in mammals, post-translational modifications (PTMs) alter and in many cases amplify the functions of proteins beyond those determined by the unmodified amino acid sequence. This greatly increases proteome complexity and influences a wide range of protein functions, including activity, solubility, folding, stability, localization, and protein–protein interactions (Aguilar-Hernández et al. 2020; Han et al. 2022; Liu et al. 2021; Vu et al. 2018). One of the most extensively studied PTMs is protein phosphorylation which provides a fast and reversible way of regulating many cellular signaling and metabolic pathways in plants (Fuente et al. 2006; Jones and Dangl 2006; Li and Liu 2021; Praat et al. 2021). Evidence from various plant species, including spinach (Zhao et al. 2018), maize (Hu et al. 2015), grape (Liu et al. 2019), and wheat (Vu et al. 2018), have demonstrated that protein phosphorylation controls the activity and the function of key components of the HS response (HSR), thereby modulating the expression of specific heat-related genes (Bourgine and Guihur 2021).

An increasing body of evidence has suggested that 3′,5′-cyclic adenosine monophosphate (cAMP) is an essential regulatory component of many processes in plants, including growth, development, and responses to environmental stresses, and it acts by modulating gene expression, protein abundance and phosphorylation (Blanco et al. 2020; Domingo et al. 2023a, b; Karimova et al. 2005; Liang et al. 2022; Paradiso et al. 2020; Xu et al. 2021; Zhao et al. 2021).

Experimental evidence has established that cAMP participates in HS tolerance by modulating the Ca^2+^ and abscisic acid (ABA) signals, modulating ROS scavenging and protein degradation, controlling vesicle trafficking, and influencing gene expression (Liang et al. 2022; Paradiso et al. 2020; Zhao et al. 2004).

Furthermore, it was recently demonstrated that, under normal temperature conditions, cAMP dampening can significantly affect an entire set of protein kinases (kinome) of tobacco BY-2 cells overexpressing a genetic tool that causes the reduction of the physiological levels of cAMP (cAS cells) (Domingo et al. 2023a, b). The analyses of differential cAMP-dependent phosphorylation identified several cAMP-dependent kinase candidates and demonstrates that the phosphorylation status of several splicing-related RNA-binding proteins is critically dependent on cAMP. This led to the central questions of this study: how does 3′,5′-cAMP affect phosphorylation during the heat response, and what insight can we gain from the understanding of heat-induced cAMP-dependent signatures?

To address this objective, a large-scale quantitative phosphoproteomics analysis was performed to assess the effects of 3′,5′-cAMP dampening on tobacco BY2 cells grown at 35 °C for 3 days. The results will be discussed with a view to provide novel insight into the role of cAMP as essential component in plant HSR and inform future research into approaches that aim at enhancing plant stress adaptation.

## Materials and methods

### Materials

Wild-type (WT) tobacco BY-2 (*Nicotiana tabacum* L. cv. Bright Yellow 2) cell suspensions were routinely propagated and cultured as described elsewhere (Nagata et al. 1992). A tobacco BY-2 line overexpressing the “cAMP sponge”, namely, cAS line (Sabetta et al. 2016), was routinely propagated in a liquid selective medium (50 µg/mL kanamycin). For the experiments, cAS cells were cultured in a non-selective medium. Two mL of both WT and cAS stationary phase cell suspensions (7 days) were diluted in 100 mL of fresh culture medium in 250 mL flasks and grown at 27 °C (control) or 35 °C (HS). After 3 days of culture, aliquots of cell suspensions were collected for the determination of cell growth and viability (Paradiso et al. 2020). Alternatively, cells were collected by vacuum filtration on Whatman 3MM paper, frozen in liquid nitrogen, and stored at −80 °C until the analyses. In total, five independent biological replicates were performed.

### Phosphoproteomic workflow

Proteins were extracted following an SDS/phenol method with minor adjustments (Wu et al. 2014). Cells (1 g) were ground with liquid nitrogen and then homogenized in Extraction Buffer (0.15 M TRIS–HCl pH 8.8, sodium dodecyl sulfate (SDS) 1%, 1 mM Ethylenediaminetetraacetic acid (EDTA), 0.1 M dithiothreitol (DTT), 1 mM phenylmethylsulfonyl fluoride (PMSF), 0.1 mg/mL Pefabloc, 1 mM Na_3_VO_4_, and 1 mM NaF). After centrifugation (15,000*g* for 10 min), the supernatant was collected and mixed with an equal volume of phenol at room temperature (RT) for 30 min. After centrifugation (15,000*g* for 5 min at RT), the phenol phase was collected, and proteins were precipitated with five volumes of 0.1 M ammonium acetate in methanol overnight at −20 °C. The protein pellet was washed once with 0.1 M ammonium acetate in methanol and once with 80% (v/v) of 0.1 M ammonium acetate in methanol. After centrifugation (15,000*g* for 5 min at 4 °C), the pellet was air dried, resuspended in SDS Lysis Buffer (Tris–HCl pH 7.5, 4% SDS, 100 mM 0.1 M), and quantified with a 2D Quant Kit (GE Healthcare, Milan, Italy).

Proteins were digested with trypsin via the Filter Aided Sample Preparation (FASP) as described previously (Vannini et al. 2019). For each sample six aliquots of proteins (200 µg each) were digested with 5 µL of trypsin porcine (conc. 1 µg/µL; Promega Italia, Milan) and at the end all the peptide fractions were collected. The peptide concentration was estimated spectrophotometrically assuming that a solution of proteins with a concentration of 1 mg/mL results in an absorbance of 1.1 at 280 nm. Peptides were desalted using Solid Phase extraction (SPE; Phenomenex Strata C18-E, Torrance, CA). All the procedures were carried out under positive pressure, except the loading and elution of the sample which made use of gravity. The column was conditioned with 3 mL 0.1% trifluoroacetic acid (TFA) in methanol and equilibrated with 2 mL Equilibration Buffer (0.1% TFA in H_2_O). After sample loading, desalting was performed at a flow rate of 1 mL of Equilibration Buffer to avoid the premature detaching of phosphopeptides. The final elution was performed by loading 1 mL of Elution Buffer (0.1% TFA in 70:30 Acetonitrile: H_2_O).

Phosphopeptides were enriched by the MagReSyn Ti-IMAC microsphere (ReSyn, Biosciences, South Africa) according to the manufacturer’s instructions. The eluted phosphopeptides were analyzed by LC-MS/MS as detailed previously (Paradiso et al. 2020).

### Data processing

Raw data were searched against the *Nicotiana tabacum* Uniprot protein database (version 2019-01, 76,141 entries) using the MaxQuant program (v.1.5.3.3) with default parameters, including Phospho (STY) in variable modifications. For the quantitative analysis the “Phospho (STY) sites” output files were processed as described by elsewhere (Domingo et al. 2023a, b).

Log_2_ transformed PP intensities were centered by the Z-score normalization method of Perseus (https://www.maxquant.org/perseus/) and subjected to a two-way ANOVA to assign statistically significant changes in PP intensity due to the treatment alone, genotype alone, and due to both treatment and genotype. In addition, ANOVA based multiple sample testing (FDR < 0.05) was followed by a post hoc test (FDR cut-off of 0.05 based on the Tukey’s test) order to discover Differentially Abundant Phosphosites (DAPPs) in WT35 vs WT27, cAS35 vs cAS27, cAS27 vs WT2 and cAS35 vs WT35 comparisons.

Hierarchical clustering was carried out using Perseus software and default parameters. The normalization of phosphoproteome data sets (Vannini et al. 2019) was conducted using the proteome background obtained previously (Paradiso et al. 2020).

The mass spectrometry proteomics data were deposited in the ProteomeXchange Consortium via the PRIDE (Perez-Riverol et al. 2019) partner repository with the data set identifier PXD040912.

### Downstream bioinformatic analyses

Log_2_ transformed and centered PP intensities were used for principal component analysis (PCA) by Perseus software in order to assess data set quality.

A local BLAST of *Nicotiana tabacum* proteins against the *A. thaliana* database (TAIR10, version 2012-05-07) was performed to use bioinformatic tools available for *A. thaliana*. Blast hits with identity < 50% and e-value > 10^–3^ were filtered out.

The enrichment analysis was performed using the Gene Ontology (GO) enrichment in Panther (December 7, 2022; www.pantherdb.org; (Mi et al. 2013) with *Nicotiana tabacum* as background. Functionally redundant terms were removed by using REVIGO (Supek et al. 2011). The proteins mapping was performed using the Kyoto Encyclopedia of Genes and Genomes (KEGG; www.genome.jp/kegg/mapper/search; Ogata et al. 1999).

The analysis of significantly enriched phosphorylation motifs was performed with the MOMO tool of the MEME suite 5.1.1 (http://meme-suite.org/tools/momo) by using the Motif-X algorithm (Cheng et al. 2019). Peptide sequences (limited to 13 amino acids) were centered on aligned modification sites (phosphoserine or phosphothreonine). The number of occurrences was set to 20, and the probability threshold was set to *p* < 10^–6^. The data set of unchanged peptides was uploaded as background data.

Kinase–target interactions were searched in the Arabidopsis Protein Phosphorylation Site Database PhosPhAt 4.0 (https://phosphat.uni-hohenheim.de) (Xi et al. 2021). Known motifs and probable kinases were searched in the PhosphoMotifFinder (http://www.hprd.org/serine_motifs) database and in the literature (Fíla et al. 2012; Marondedze et al. 2016; Mayank et al. 2012; van Wijk et al. 2014).

The scanning for occurrences of putative cyclic nucleotide-binding motifs and PKA kinase consensus motif (…RR-X-S/T…) was done by using Find Individual Motif Occurrences (FIMO; MEME suite 4.11.4) with match p-value lower than 1e10^−4^ (Grant et al. 2011). The cyclic nucleotide-binding domain signatures (PS00888; PS00889) obtained from the Prosite database of protein domains (https://prosite.expasy.org/PDOC0069) were used. The generation of sequence logos was done using the web-based application WebLogo (https://weblogo.berkeley.edu/).

### Molecular docking analysis

Models of the 3D structure of the protein of interest have been produced using the Swiss-Model server (Waterhouse et al. 2018). Models showing the highest overall QMEANDisCo score were used. Regions of the proteins with a 3D model confidence score (based on local QMEANDisCo or pLDDT score) < 0.50 were not considered for docking simulations. Docking simulations were performed using Autodock Vina (Trott and Olson 2010) and analyzed using PyMol (The PyMOL Molecular Graphics System, Version 2.0 Schrödinger, LLC). Docking analyses were performed considering the whole space occupied by the protein as the searching grid (i.e., according to a blind docking procedure).

### Phosphosite conservation analysis

Phosphosite conservation analysis was conducted as detailed previously (Van Leene et al. 2019). Essentially, a multiple sequence alignment (MSA) with each phosphoprotein and its paralogues was performed. For each protein, the best BLAST hit and its paralogues in reference organisms (*A.* *thaliana*, *B.* *rapa*, *E. grandis*, *G. max*, *P. trichocarpa*, *V. vinifera*, *S. lycopersicum*, *O.* *sativa* ssp. Japonica, *A.* *trichopoda*, *P.* *patens*, and *C. reinhardtii*) were selected by using PLAZA 5.0 dicots (Proost et al. 2015). Conservation of the residues, as well as the flanking sequences (window) around the residues (− 6 or + 6), was determined by remapping all residue positions within the *N. tabacum* protein. The percentage of conserved phosphosites was calculated for every species where the phosphorylated residue was present. The BLOSUM score was used to score the conservation of the sequence within the window around the residue.

### PPI network construction and essential protein/hub analysis

The Search Tool for Retrieval of Interacting Genes (STRING) database (https://string-db.org) was used to point to potential interactions between all phosphoregulated proteins in cAS vs. WT comparisons (Szklarczyk et al. 2019). Parameters were set as follows: co-expression as active interaction sources and medium confidence (> 0.4). Disconnected nodes were hidden in the network. In order to visualize the protein–protein interaction (PPI) network the Cytoscape software version 3.6.1 was used (Shannon et al. 2003). The maximal Clique Centrality (MCC) algorithm of the CytoHubba plugin (Chin et al. 2014) was used to detect the top hub genes in co-expression networks. Proteins with the top MCC values were considered hub genes/proteins.

## Results and discussion

### The effect of cAMP on Differential Abundance of Phosphosites (DAPPs) during heat stress

HS at 35 °C slows the growth of tobacco BY-2 cells, due to an inhibition of cell division after 3 days and a progressive reduction of cell expansion and viability (Centomani et al. 2015). Cyclic AMP-deficient (cAS) lines of tobacco BY-2 cells have been shown to be more sensitive to prolonged HS than wild-type (WT), showing a higher decrease in cell growth after 3 and 5 days of growth at 35 °C, principally due to increased cell death (Paradiso et al. 2020).

To elucidate how different mechanisms of HSR are influenced by cAMP levels, we investigated the phosphoproteomic changes of both WT and cAS lines of tobacco BY-2 cells grown for 3 days at 35 °C. Under these conditions the HS already impairs different biological processes but does not yet severely affect cell growth and viability (Centomani et al. 2015; Paradiso et al. 2020).

Using a Ti-IMAC microsphere-based enrichment proteomic approach, we identified 3531 phosphosites distributed on 2979 unique peptides, mapping to 1958 proteins, derived from four biological replicates (Fig. S1A). Principal Component Analysis (PCA) demonstrates reproducible quantitative values among replicates and reveals a clear distinction among all samples, indicating that both genotype and temperature regimes influence the BY-2 proteome (Fig. S1B). The majority of the identified phosphosites were serine residues (over 80%), followed by threonine residues at approximately 13%, with tyrosine residues constituting less than 1% and this is consistent with previous reports in *A. thaliana* (Sugiyama et al. 2008).

The one-way ANOVA comparison (FDR < 0.01) allowed us to identify 411 Differentially Abundant Phosphosites (DAPPs) across the four analytical groups (Table S1). To gain further insight into the influence of genotype, temperature, and their interaction on changes in the phosphoproteome, we conducted a two-way ANOVA analysis on DAPPs (Fig. S1A). This analysis reveals that 143 DAPPs are affected by cAMP depletion (genotype-dependent list). Another 177 DAPPs are influenced by HS (temperature-dependent list) and 180 DAPPs are influenced by the genotype–temperature interaction (genotype–temperature interaction-dependent list) (Tables S2–4).

For 935 phosphosites (PPs) we quantified the matching proteins (455) in the proteome background experiment published previously (Paradiso et al. 2020). This allowed for normalization of phosphosite intensities based on the abundance of the corresponding total amount of each protein. The ANOVA comparison test (FDR < 0.051) revealed 48 PPs with changing degrees of phosphorylation (Table S5) and led to the identification of 9 potentially critical phosphosites that could not have been detected as differentially abundant without the normalization procedure (Table S5).

Overall, these data suggest that cAMP markedly impacts the phosphoproteome in response to HS. This implies that kinases and phosphatases are likely to be to be activated or inactivated by high temperature resulting in distinct cAMP-dependent phosphoforms of target proteins.

### Protein phosphorylation motifs associated with temperature and cAMP-dependent temperature responses

The analysis of temperature-dependent and genotype–temperature interaction lists reveals significant enrichments of (S/T)-P motifs in both up-regulated and down-regulated phosphosites (Fig. S1C). These motifs are well-established targets for various kinases, including mitogen-activated kinases (MAPKs), receptor-like kinases (RLKs), cAMP-dependent or cGMP-dependent protein kinases (AGCKs), cyclin-dependent kinases (CDKs), sucrose non-fermenting 1-related protein kinases (SnRKs), NIMA-related kinases (NEKs), and calcium-dependent kinases (CPKs) (van Wijk et al. 2014). Consistent with these observations, we noted differential phosphorylation of kinases belonging to the families modulated by both HS and cAMP-HS interactions (Table S6). Predicted protein kinases capable to phosphorylate targets in the HS and cAMP-HS interaction data sets have been retrieved from the Arabidopsis Protein Phosphorylation Site Database (Table S7).The most prevalent kinase families again point to a key roles of MAPKs and CDKs, both critical components of HSR in *Lycopersicon esculentum*, a Solanaceae species similar to tobacco (Ding et al. 2018; Hu et al. 2021).

Interestingly, several proteins of the cAMP-HS interaction data set have been identified as potential targets of the AGCK family (Fig. S2). Since plants share AGC family members with other eukaryotes but lack the typical PKA and PKC subfamilies (Rademacher and Offringa 2012), we speculate that they may have evolved different AGCK subfamilies for cAMP-dependent signaling during HS. It is noteworthy that the PKA kinase consensus motif (…RR-X-S/T…) occurred in 10 phosphoregulated proteins of the interaction data set (Table S8), supporting the idea that PKAs with no orthologues in animals may exist in plants. Among them are two serine/arginine repetitive matrix proteins (A0A1S4AYR8, A0A1S3X572) and the serine/arginine-rich SC35-like splice factor SCL33 (A0A1S3YID9).

### In search of candidate cAMP-binding protein kinases

To gain further insight into cAMP–protein interactions, proteins of the interaction list were assessed for the presence of two cyclic nucleotide-binding domain signatures (PS00888, PS00889; https://prosite.expasy.org/PDOC0069). The domain signature 1 (PS00888) was detected in 9 phosphoregulated proteins, while the domain signature 2 (PS00889) was found in 19 phosphoproteins (Table 1).

**Table 1:** DAPPs containing putative cyclic nucleotide-binding domains

Cyclic nucleotide-binding domain PS00888							
Alt ID	ID	Description	Ara ID	Start	End	*p*-value	Matched sequence
XXXHGXXXLXXXG	A0A1S3YCC6	Serine/threonine-protein kinase HT1-like	AT3G22750.1	103	115	1.74E−05	LVAKGTYGTVYRG
XXXHGXXXLXXXG	A0A1S4BQJ5	Serine/threonine-protein phosphatase	AT2G27210.1	788	800	2.36E−05	ICMHGGIGRSINH
XXXHGXXXLXXXG	A0A1S4CPD1	Uridine kinase	AT5G40870.1	314	326	5.37E−05	VVEHGLGHLPFTE
XXXHGXXXLXXXG	A0A1S3ZL27	Serine/threonine-protein kinase ATG1c-like	AT2G37840.1	540	552	6.47E−05	DICHTQAASAIEG
XXXHGXXXLXXXG	A0A1S4A7G6	DEAD-box ATP-dependent RNA helicase 37-like	AT2G42520.1	432	444	7.35E−05	LCINGFPATAIHG
XXXHGXXXLXXXG	A0A1S4CPK5	Uncharacterized protein LOC107821022	AT2G35050.1	1156	1168	9.47E−05	ICKVGDFGLSKIK
XXXHGXXXLXXXG	A0A1S4DLC7	Uncharacterized protein LOC107830986 isoform X2	AT1G17210.1	878	890	9.59E−05	IKHHNFFCPWVNG
XXXHGXXXLXXXG	A0A1S3XBA1	Flowering time control protein FPA-like isoform X2	AT2G43410.3	802	814	9.77E−05	SGTHSADALGLYG
XXXHGXXXLXXXG	A0A1S4CNE1	Phosphatidylinositol 4-kinase alpha 1	AT1G49340.1	1389	1401	3.39E−05	LCQHEADRLDVWA

Cyclic nucleotide-binding domain PS00889							
Alt ID	ID	Description	Ara ID	Start	End	*p*-value	Matched sequence
XGEXXXXRXAXXXX	A0A1S3ZL94	Probable alkaline/neutral invertase D	AT4G09510.1	193	206	7.84E−08	FGESAIGRVAPVDS
XGEXXXXRXAXXXX	A0A1S4BQJ5	Serine/threonine-protein phosphatase	AT2G27210.1	144	157	8.34E−07	IGEPPTPRAAHVAT
XGEXXXXRXAXXXX	A0A1S4AB39	Enhancer of mRNA-decapping protein 4-like	AT3G13300.2	742	755	1.65E−06	VGEYSVDRQMDAIH
XGEXXXXRXAXXXX	A0A1S4BWE6	Topless-related protein 4-like isoform X3	AT3G15880.1	403	416	1.34E−05	LGQCSVALQASLAS
XGEXXXXRXAXXXX	A0A1S4BFG6	Transcriptional corepressor LEUNIG_HOMOLOG-like isoform X2	AT2G32700.6	502	515	2.69E−05	FGEVGCIRTRNKVT
XGEXXXXRXAXXXX	A0A1S3YG89	Uncharacterized protein LOC107775706	#N/D	505	518	2.81E−05	VGMVAVAAAAAAAA
XGEXXXXRXAXXXX	A0A1S3YCC6	Serine/threonine-protein kinase HT1-like	AT3G22750.1	129	142	3.55E−05	WGEDGMATAAETAA
XGEXXXXRXAXXXX	A0A1S4A022	Golgin candidate 2-like isoform X2	AT1G18190.1	289	302	4.30E−05	LDENKRIRSAKAMV
XGEXXXXRXAXXXX	A0A1S4D427	Condensin-2 complex subunit D3-like isoform X1	AT4G15890.1	443	456	5.65E−05	MDEKAAVRKAALLV
XGEXXXXRXAXXXX	A0A1S4CCN5	Splicing factor, arginine/serine-rich 19-like	AT1G36990.1	211	224	5.75E−05	IGSISMGSSASQHS
XGEXXXXRXAXXXX	Q1ZZN8	Pollen tube RhoGDI2	AT3G07880.1	28	41	6.39E−05	VGEKNVSRQMSESS
XGEXXXXRXAXXXX	A0A1S3ZUH7	Condensin complex subunit 2-like isoform X1	AT2G32590.1	28	41	6.50E−05	KLERARARAARAAV
XGEXXXXRXAXXXX	A0A0D4D8G6	Auxin efflux carrier component	AT5G57090.1	161	174	7.35E−05	IGEQFPETAASITS
XGEXXXXRXAXXXX	A0A1S4D427	Condensin-2 complex subunit D3-like isoform X1	AT4G15890.1	298	311	7.85E−05	APEKAEARAAAVEA
XGEXXXXRXAXXXX	A0A1S3Z2A7	DnaJ homolog subfamily C GRV2-like isoform X3	AT2G26890.1	1135	1148	8.85E−05	SGEPSVVESAAALL
XGEXXXXRXAXXXX	A0A1S4CIX0	Auxilin-like protein 1	AT4G36520.1	284	297	8.98E−05	IGKEGHARTGDLHV
XGEXXXXRXAXXXX	A0A1S4A262	Uncharacterized protein LOC107792916 isoform X1	AT2G02170.1	251	264	9.61E−05	MIENSIGQSAINLS
XGEXXXXRXAXXXX	A0A1S3ZXT8	Uncharacterized protein LOC107791595	AT1G05380.2	318	331	9.75E−05	VKEESMSAAAEDVT
XGEXXXXRXAXXXX	A0A1S4DQV8	GBF-interacting protein 1-like isoform X4	AT5G48940.1	164	177	8.85E−05	FGSNSVVHDAHASA

The scanning for occurrences of putative motifs was done by using Find Individual Motif Occurrences (FIMO; MEME suite 4.11.4) searching for cyclic nucleotide-binding domain signatures obtained from the Prosite database (PS00888 and PS00889; https://prosite.expasy.org/PDOC0069)

**Fig. 1:**
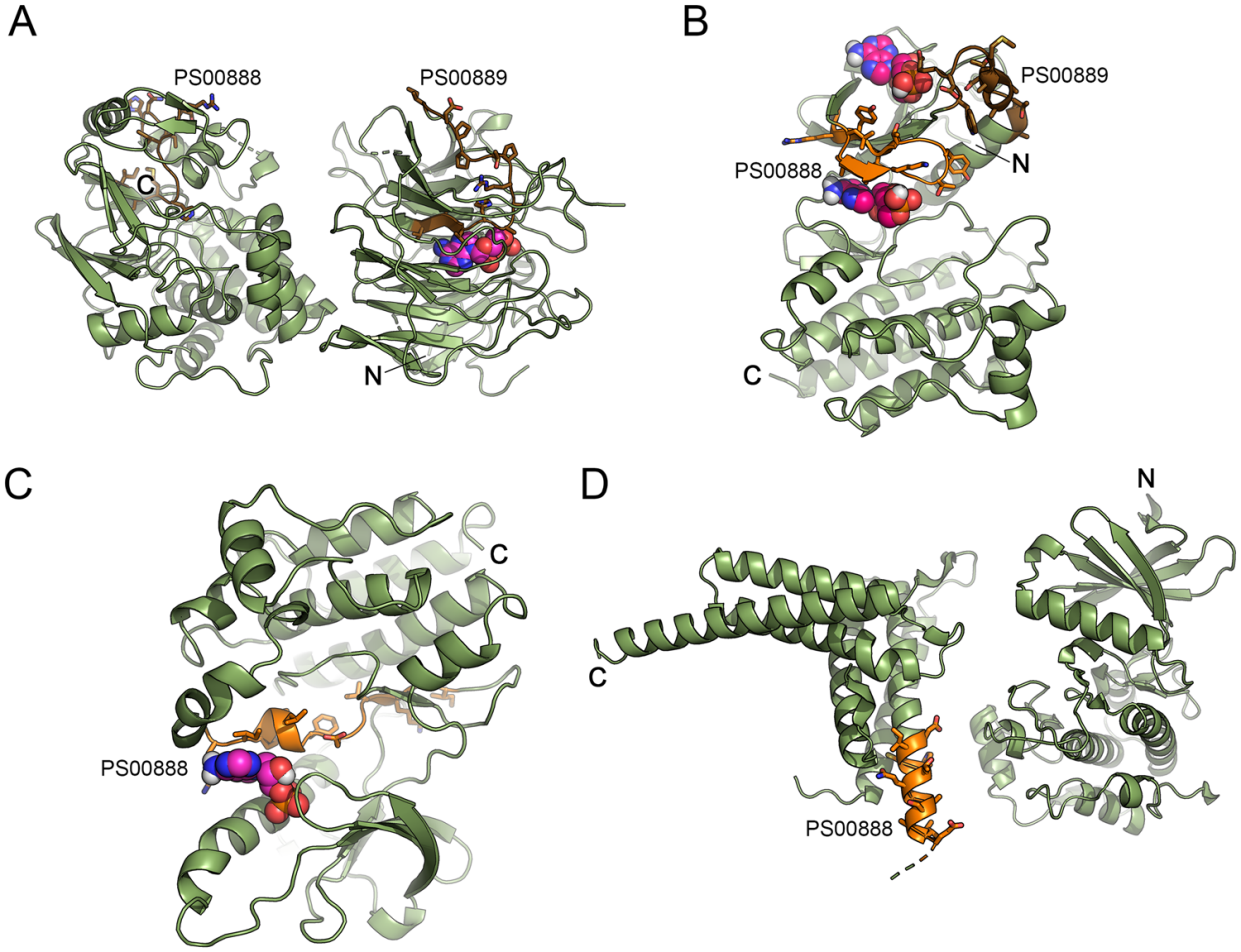
Molecular docking analysis showing the predicted docked poses of cAMP with the proteins of interest. **A** Predicted complex between cAMP and A0A1S4BQJ5. Left, C-terminal domain with PS00888 signature; right, N-terminal domain with PS00889 signature. The docked poses have been individually predicted by two independent docking simulations. **B** Predicted complex between cAMP and A0A1S3YCC6. The two shown poses represent solutions 1 and 5 of the docking simulation. **C** Predicted complex between cAMP (solution 1 of the docking simulation) and A0A1S4CPK5. The protein N-terminal is behind the ligand. **D** Model of the 3D structure of A0A1S3ZL27. Regions of the proteins whose 3D model confidence score was < 0.50 were not considered. The cAMP molecules are represented as spheres. The domain signatures PS00888 and PS00889 are represented in yellow and brown, respectively

Even if cAMP-dependent kinases and phosphatases have not currently been identified in plants, their likely involvement in cAMP-dependent signaling has been proposed (Domingo et al. 2023a, b; Zhao et al. 2004), and this study lends further support to the presence of plant cAMP-dependent kinases. Encouragingly, both cyclic nucleotide-binding domain signatures were found within the sequence of the phosphatase A0A1S4BQJ5. Incidentally, the *A. thaliana* orthologue, the BRI1 suppressor 1-like 3 (BSL3; AT2G27210), is implicated in brassinosteroid signaling (Mao and Li 2020). The serine/threonine-protein kinase ATG1c-like (A0A1S3ZL27) that plays a role in autophagy (Meijer et al. 2007) also has a 13 residue-long candidate cAMP-binding site between positions 540 and 552. This location is right next to the serine (S553) that showed increased phosphorylation in cAS cells under HS. Moreover, the kinases A0A1S3YCC6 and A0A1S4CPK5 have their putative cAMP-binding residing within the protein kinase domain. We therefore propose that the phosphorylation status of these proteins might be linked to cAMP binding and hence the modulation of kinase activity. In an attempt to further support such a proposed functional link between cAMP and the phosphorylation status, the potential ability of the 3 putative kinases (A0A1S3ZL27, A0A1S3YCC6, and A0A1S4CPK5) and of the putative phosphatase (A0A1S4BQJ5), which possess the cyclic nucleotide-binding domain signatures in their sequences, was investigated through molecular docking simulations. In the case of A0A1S4BQJ5, which is predicted to be a two-domain protein, simulation suggests that cAMP can be bound by the protein in a region of the N-terminal domain close to the PS00889 site and in a region of the C-terminal domain close to the PS00888 site, and in both cases an identical predicted binding energy of −8.2 kcal/mol) (Fig. 1A). The putative serine/threonine-protein kinase A0A1S3YCC6 in turn also harbors both Cyclic Nucleotides Monophosphate (cNMP) binding sequences even though it is a single-domain protein.

Docking analysis also confirmed the feasibility of cAMP binding at positions in the close vicinity of the binding sequences with binding energies of −7.3 kcal/mol for the site PS00888 and −6.0 kcal/mol for PS00889 (Fig. 1B). On the other hand, A0A1S4CPK5, another single-domain protein, possesses only the PS00888 cNMP binding motif to which, accordingly to docking simulations, cAMP can bind with a predicted binding energy of −7.7 kcal/mol (Fig. 1C). Finally, it was not possible to build a reliable 3D model of the putative two-domain serine/threonine-protein kinase A0A1S3ZL27 (Fig. 1D). For this reason, docking analysis was not performed on the latter. It should be emphasized that all docking analyses were performed considering the whole space occupied by the protein as the searching grid (i.e., according to a blind docking procedure), thus further minimizing any bias due to the prior knowledge of the position of the predicted binding motifs. Overall, the predicted binding energies range from −6.0 to −8.2 kcal/mol, an interval that agrees with the expected ones for compounds in that molecular mass range. Overall, the predicted structural binding regions coincide with those the motif-based prediction, thus supporting the idea that cAMP can conceivably bind at these sites enabling direct interactions with residues of the motifs. Taken together, these findings are consistent with an interaction of cAMP with target kinases and/or phosphatases in modulating HSR.

### Specific temperature-dependent changes in the phosphorylation profile

**Fig. 2:**
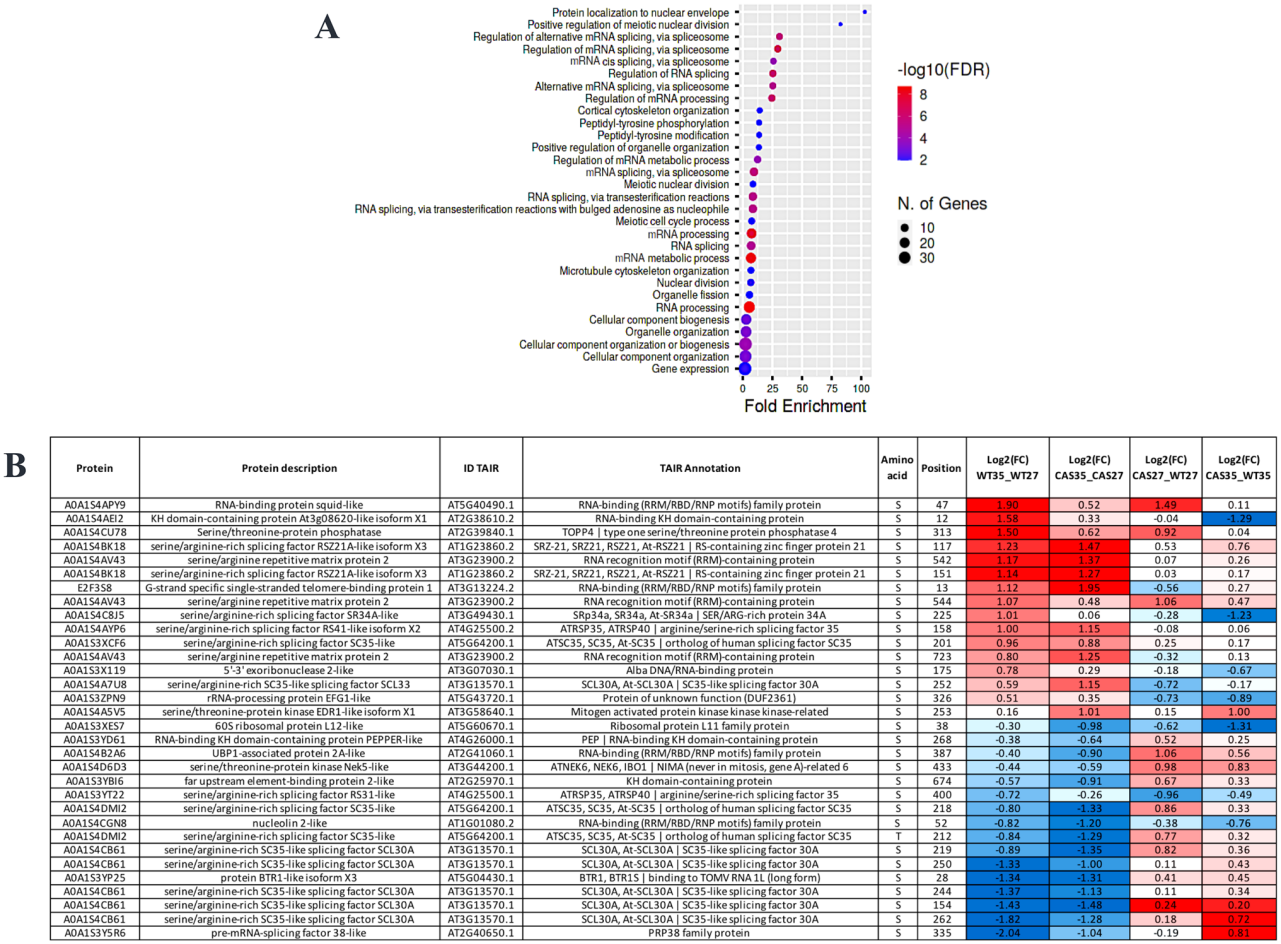
**A** Gene ontology statistically overrepresented categories in temperature data set calculated using Panther (http://www.pantherdb.org/) and the *Nicotiana tabacum* as background. Functionally redundant terms were removed with REVIGO. **B** List of phosphoregulated proteins involved in RNA processing and splicing with their respective phosphosites and log fold change values

To further investigate the biological processes associated with HS, we characterized the phosphoproteins of the temperature-dependent list by using a GO analysis. Among the most enriched biological processes were “gene expression (GO:0010468)”, “RNA processing (GO:0006396)”, “RNA splicing (GO:0008380)”, “organelle organization (GO:0006996)”, “nuclear division (GO:0000280)”, and “cortical cytoskeleton organization (GO:0030865)” (Fig. 2A). RNA binding is the most overrepresented molecular function. RNA-binding proteins (RBPs) recognize and specifically bind target RNA sequences, thereby modulating activity and fate of transcripts (Marondedze et al. 2019). Moreover, RBPs also have a been established as functional components of abiotic stress response processes (Yan et al. 2022).

**Fig. 3:**
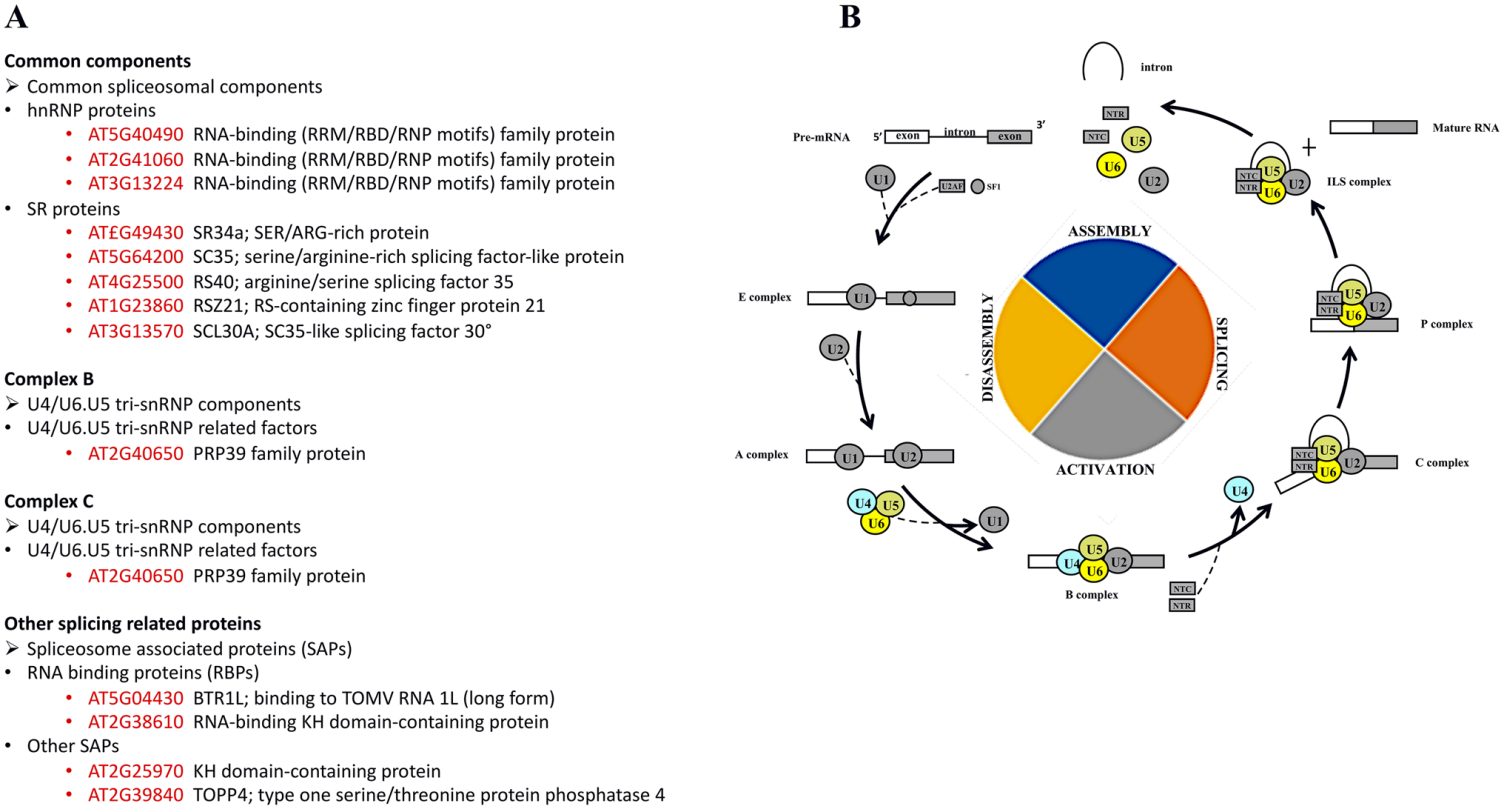
**A** Splicing-related proteins mapped using the Kyoto Encyclopedia of Genes and Genomes (KEGG; https://www.genome.jp/kegg/mapper/search.html). **B** Schematic representation of the spliceosome activation process including the phosphoregulated proteins (colorful shapes)

Our study reveals significant differential phosphorylation of 32 DAPPs in 24 phosphoproteins with a role in mRNA splicing (Fig. 2B). In particular, serine/arginine-rich (SR) splicing factors seem affected, many of which are annotated as operating in spliceosome activation (Fig. 3). Our analysis also revealed temperature-dependent phosphorylation of the PK12 kinase (O49967) which has an orthologue in tobacco that is implicated in SR protein phosphorylation (Golovkin and Reddy 1999; Savaldi-Goldstein et al. 2000). We therefore speculate that this differential phosphorylation is implicated in the phosphorylation status changes in SR proteins.

Protein–Protein Interaction (PPI) network analysis of phosphoregulated proteins responding to high temperature delineates two distinct networks with proteins involved in ribosome biogenesis/gene expression and RNA processing/splicing, respectively (Fig. S3A,B). Notably, most connected proteins (hubs) within these networks included the nucleolar GTP-binding protein 1 (NOG1; A0A1S4D9M3; AT1G50920) that is essential for ribosomal biogenesis as well as being responsive to stress (Lee et al. 2018). Also highly connected is the serine/arginine-rich splicing factor SC35 (A0A1S3XCF6; A0A1S4DMI2; AT5G64200) that shows decreased the phosphorylation of T212 and S208 and increased phosphorylation of S201. The tool PhospPPI (https://phosppi.sjtu.edu.cn/; (Hong et al. 2023) predicts that phosphorylation at S201 increases the SC35 interaction with its interactor, SCL30A (Yan et al. 2017). By comparing proteins of the temperature-dependent data set with those phosphoregulated by HS in different plant species (Liu et al. 2019; Vu et al. 2021; Zhang et al. 2020; Zhao et al. 2018), we noted that 34 proteins have *A. thaliana* orthologues that are also differentially phosphorylate under HS in at least one other plant species. Incidentally, proteins with shared HS-dependent phosphorylation include the SR splice factors SC35, SCL30A, and SR40 (Table S9).

In conclusion, our results are consistent with data in the literature that propose that alternative splicing may act as a “molecular thermometer” enabling plants to adapt transcript abundance in response to temperature increases (Capovilla et al. 2015; Dikaya et al. 2021; Liu et al. 2022) and that this process is tightly regulated by protein phosphorylation.

### cAMP participates in mRNA metabolism under heat stress

**Fig. 4:**
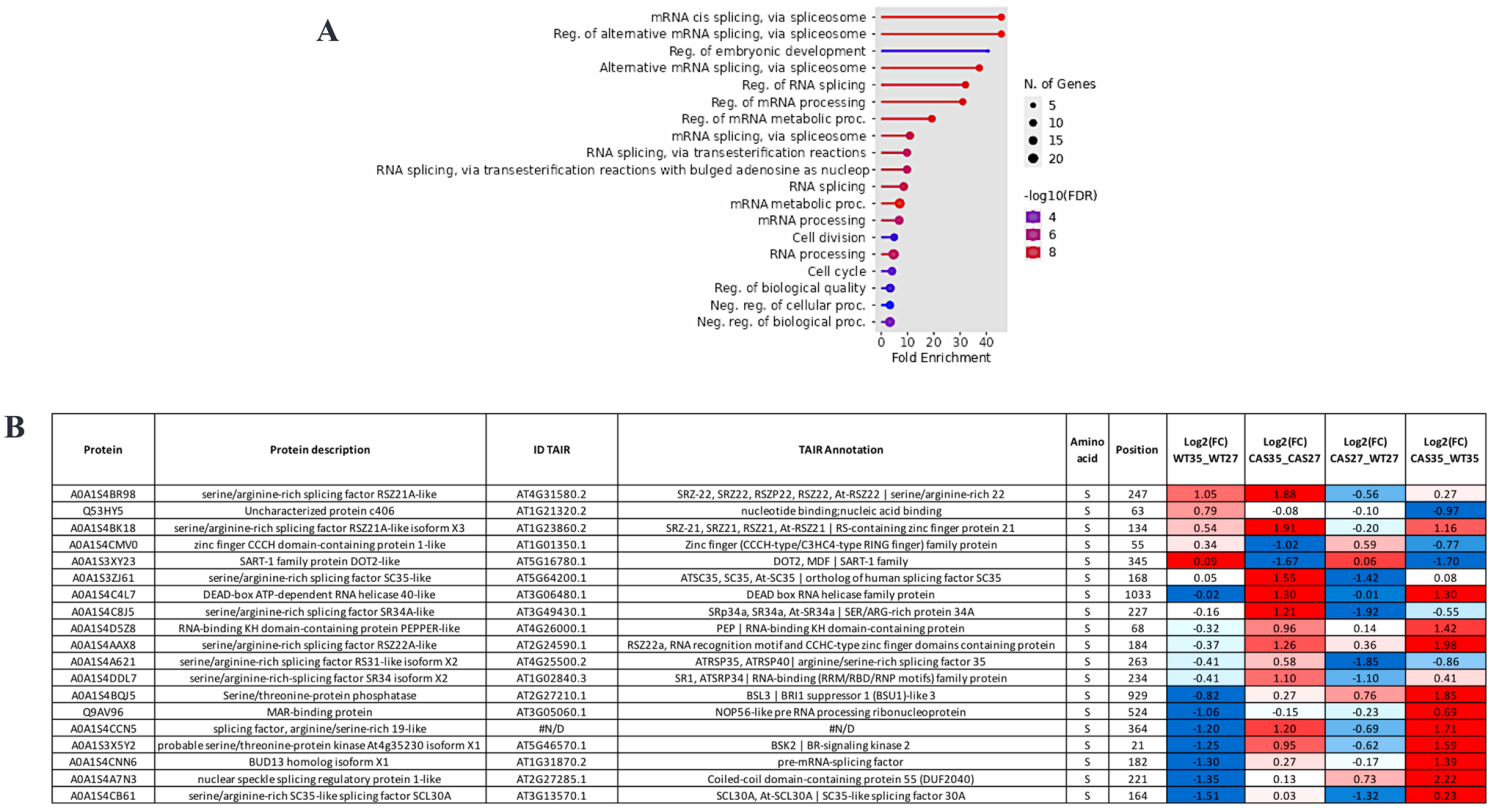
**A** Gene ontology statistically overrepresented categories in interaction data set calculated using Panther (http://www.pantherdb.org/) and the *Nicotiana tabacum* as background. Functionally redundant terms were removed with REVIGO. **B** List of phosphoregulated proteins involved in RNA processing and splicing with their respective phosphosites and log fold change values

**Fig. 5:**
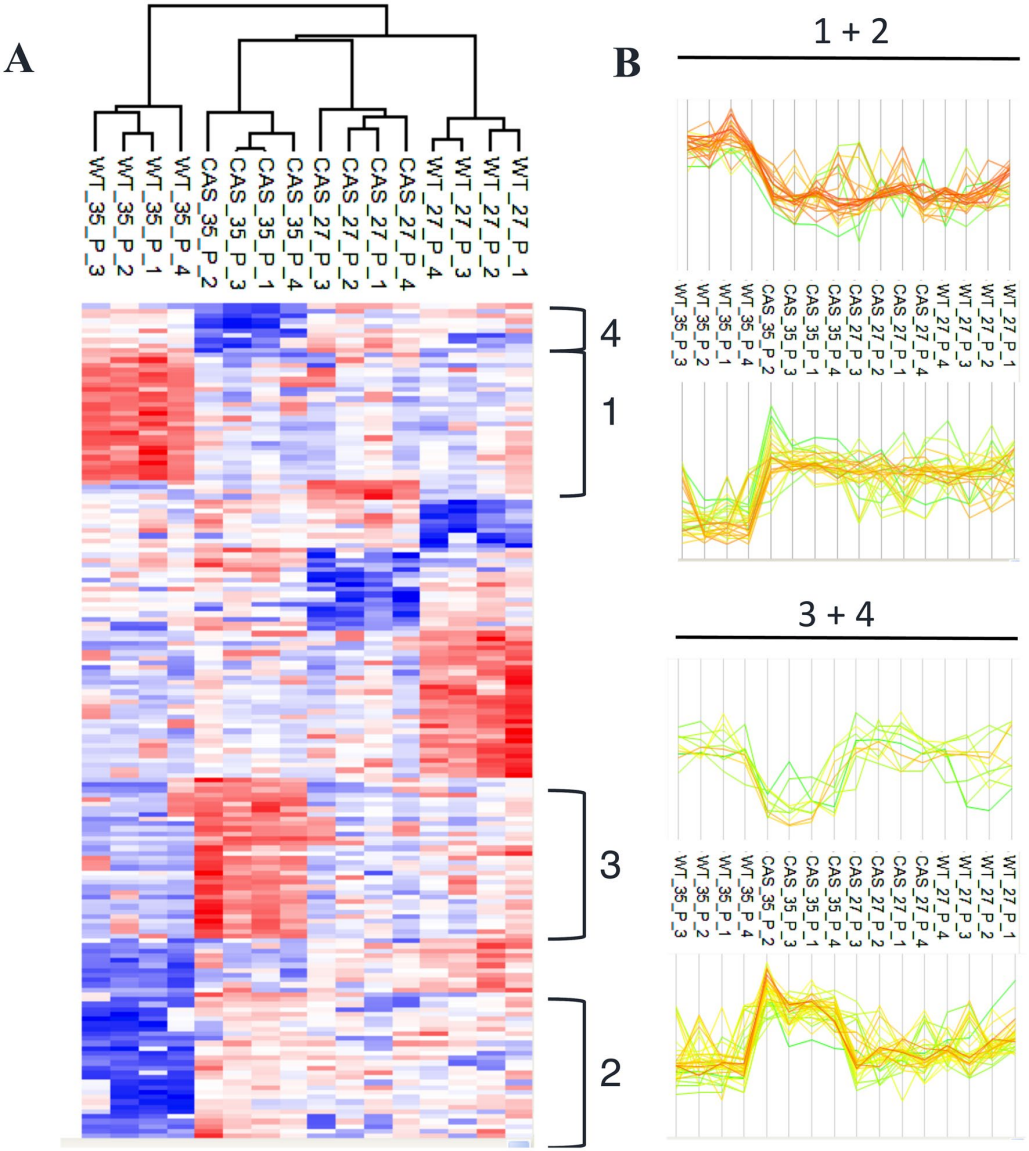
**A** Hierarchical clustering analysis of the interaction data set carried out using Perseus software. **B** Selected clusters of DAPPs responding to HS exclusively in WT (clusters 1, 2) and cAS cells (clusters 3, 4)

The GO analysis of the 160 differentially phosphoregulated proteins of the genotype–temperature interaction list points to a critical function of cAMP in the phosphorylation of numerous proteins involved in RNA processing and splicing, including 19 RBPs (Fig. 4). For a more in-depth analysis of the specific phosphorylation changes in the two genotypes (WT and cAS cells) in response to HS, the DAPPs of this list were clustered. Figure 5 shows some of the phosphorylation profiles obtained. Clusters 1 and 2 consist of 26 and 35 proteins that are hyper- of hypo-phosphorylated exclusively in the presence of cAMP in response to HS (Fig. 5A, B; Table S10). They therefore represent HS-responsive elements that are not regulated in the absence of cAMP. Among them are RNA splicing components (A0A1S4CNN6, Q53HY5, A0A1S4A7N3) and in particular, the DAPP (S182) in A0A1S4CNN6 which is a highly conserved phosphosite in 11 different plant species considered (Table S11). Incidentally, A0A1S4CNN6 has an orthologue in *A. thaliana* (BUD13; AT1G31870) that has a well-documented role in the pre-mRNA splicing of several genes involved in nucleic acid metabolism and development (Xiong et al. 2019). Additionally, nucleic acid/nucleotide-binding protein (NSRB; AT1G21320), the orthologue of Q53HY5 in *A. thaliana*, physically interacts with WRKY25 and WRKY33 (https://thebiogrid.org/23968/summary/arabidopsis-thaliana/at1g21320.html), both of which are transcription factors enabling plant thermotolerance (Cheng et al. 2012; Li et al. 2011).

Several cAMP-dependent HS-responsive RBPs are also involved in mRNA degradation, a process that is vital for mRNA homeostasis in response to environmental stressors. Among them are the la-related protein 1C-like (A0A1S4BFV1), the FPA-like isoform X2 (A0A1S3XBA1), and the pumilio homolog 4-like (A0A1S4A3Z4), all of which contribute to the rapid degradation of mRNAs under stress (Merret et al. 2013; Sun et al. 2017). We also observed increased phosphorylation of the general negative regulator of transcription subunit 3 isoform X2 (A0A1S4CSL2) and the enhancer of the mRNA-decapping protein 4-like (A0A1S4AB39), both of which participate in RNA decay in cytoplasmic foci referred to as processing bodies (Maldonado-Bonilla 2014). Finally, the *A. thaliana* homologs of A0A1S4AB39 and A0A1S4CPK5, VCS (AT3G13300), and Raf24 (AT2G35050) have all been implicated in mRNA degradation during osmotic stress (Soma et al. 2020). Interestingly, three of these proteins (A0A1S4AB39, A0A1S4CPK5, A0A1S3XBA1) harbor cAMP-binding motifs (Table 1), thus further supporting a direct role of cAMP in the post-transcriptional regulation of proteins involved in the mRNA turnover during prolonged HS.

The data also highlight the role of cAMP in regulating transcription during HSR since it increases phosphorylation of the cAMP-dependent Mediator complex subunit MED14 (A0A1S3Z923). The *A. thaliana* orthologue of the latter (AT3G04740) has a critical role in the organization of the mediator complex and cooperates in activating heat stress-inducible genes (Hemsley et al. 2014; Ohama et al. 2021).

In addition, phosphorylation of the SWR1-complex protein 4-like (SWC4, A0A1S4CD96) at the highly conserved phosphosite T389 decreases only in WT cells during HS and this is significant since the cAMP-dependent phosphorylation site is situated in the DMAP1 domain which mediates protein–protein interactions (Zhou et al. 2010). In *A. thaliana,* SWC4 is associated with the SWR1 and the NuA4 acetyltransferase complexes which both have a role in the epigenetic regulation (Espinosa-Cores et al. 2020; Willhoft and Wigley 2020). Recently, it has been demonstrated in pepper that SWC4 affects the responses to both pathogen infection and HS and does so by modulating chromatin of specific target genes (Cai et al. 2022). We therefore propose that cAMP-dependent phosphorylation of SWC4 may contribute to altered responses of the cAMP-depleted cells, both in response to HS and pathogens (Paradiso et al. 2020; Sabetta et al. 2019).

### cAMP mediates phosphorylation of proteins implicated in cell cycle progression during heat stress

The impact of stress on plant growth requires complex coordination of physiological processes to maintain homeostasis (Bechtold and Field 2018). Heat stress significantly impacts cell division by slowing cell cycle progression (Qi and Zhang 2019). It is therefore not surprising that we identified several differentially phosphorylated proteins involved in cell cycle progression that may link phosphorylation to temperature-stress and cAMP levels. Among proteins affected by HS only in the presence of cAMP (Table S10-clusters 1, 2), we found two condensins (A0A1S4D427, A0A1S3ZUH7) reported to be phosphorylated during mitosis and then dephosphorylated upon the completion of the mitotic cycle (Takemoto et al. 2004). These proteins too contain cAMP-binding motifs which is further support for a pivotal role of cAMP in their phosphorylation and their role in cell cycle regulation during heat stress.

Cyclic AMP-dependent decreases in phosphorylation occurs also in several S6 ribosomal proteins (RPS6; A0A1S3YPM6, A0A1S4BZ85, A0A1S3YRP9; Table S10-cluster 2) which have been associated with the decrease in mitotic index during heat stress in tomato cells (Scharf and Nover 1982).

The clusters 3 and 4 (Fig. 5, Table S10) contain 35 differentially phosphorylated proteins responding to HS exclusively in cAS cells. This cAMP dependence underlines the critical role of this messenger in the cellular adaptation to HS conditions. Among those proteins, several are in the GO categories “cellular response to stimulus (GO:0051716)”, “RNA processing (GO:0006396)”, and “programmed cell death (GO:0012501)”. The category “programmed cell death” includes a remorin (A0A1S4A262) which, when overexpressed in *N. benthamiana,* triggers cell death by increasing accumulation of reactive oxygen species (Cai et al. 2020). It is noteworthy that this remorin too contains a cAMP-binding motif. Finally, LAZ1 (A0A1S3YMD8) which operates in vacuolar transport and is impacting brassinosteroid and programmed cell death signaling pathways (Liu et al. 2018) also exhibits altered phosphorylation signatures (Table S10-cluster 3). These observations therefore also tie decreases in the mitotic index during heat stress to an essential role of cAMP (Paradiso et al. 2020).

## Conclusion

A growing body of evidence is emphasized the role of cAMP in stress response signaling (Blanco et al. 2020). Specifically, an increase in free 3′,5′ cAMP in response to HS has been found to be a crucial component of HSR in tobacco BY-2 cells (Paradiso et al. 2020). However, the elucidation of the underlying molecular mechanisms of cAMP-dependent processes during HSR remain fragmentary. In turn, protein phosphorylation and dephosphorylation have been well established as having profound effects on protein structure, activity, subcellular distribution, and interaction with other proteins, ultimately influencing plant growth, and stress responses. Our aim here was to analyze phosphoproteomic changes during HSR and delineate the role of cAMP in this process. To this end we studied the phosphoproteomic changes during HSR in a cAMP-depleted cell line which has been shown to be more sensitive to prolonged HS (Paradiso et al. 2020). The findings establish a systemic role of cAMP as modulator of the phosphorylation status of many proteins and notably proteins that function in mRNA processing, transcriptional control, and cell cycle regulation under HS. In particular, RBPs are highly enriched among cAMP-dependent HS responsive phosphoproteins, consistent with a role of cAMP as direct or indirect modulator of RBP function during HS. In addition, both motif scan and molecular docking investigations suggest the presence of candidate cAMP-dependent protein kinases and phosphatases able to directly interact with cAMP.

Taken together, our findings shed light on the complex interactions between cAMP signaling and HSR mechanisms, showing that cAMP-dependent phosphorylation is essential for high-temperature adaptation. Moreover, this study also proposes candidates for in-depth studies of mechanisms that underlie plant adaptation to stress in general and heat stress in particular.

### Supplementary Information

Below is the link to the electronic supplementary material.

Supplementary file1 (XLSX 546 KB)

Supplementary file2 (TIF 4133 KB)

Supplementary file3 (TIF 2551 KB)

Supplementary file4 (TIF 2163 KB)

## Data Availability

The data that support the findings of this study are available from the corresponding author upon reasonable request.
