# Supplementary figures and images for: The cAMP-dependent phosphorylation footprint in response to heat stress
Source: Plant Cell Rep. 2024 May 7;43(6):137. doi: 10.1007/s00299-024-03213-y (PMC11076351; doi:10.1007/s00299-024-03213-y)

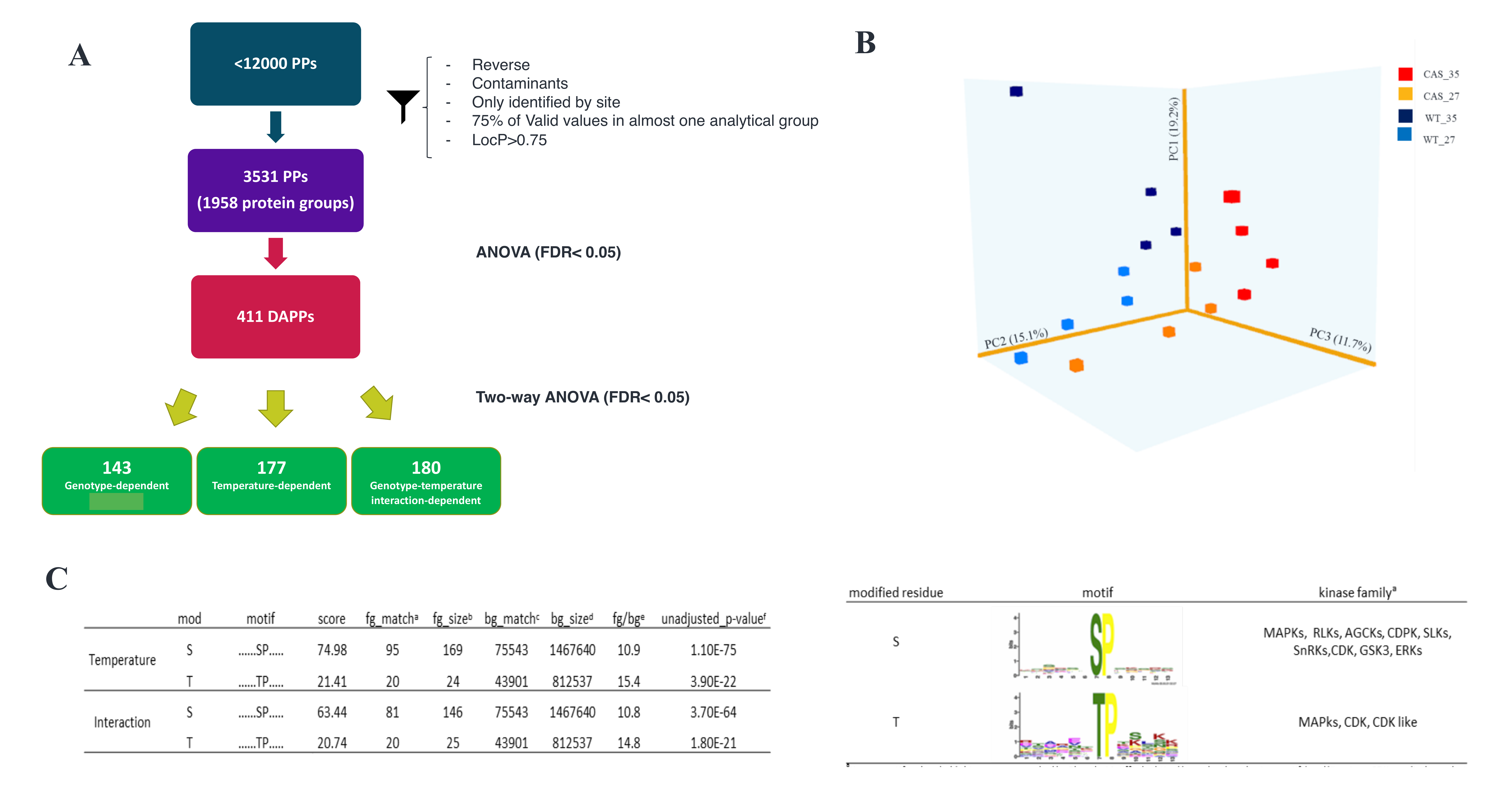

Supplement: Supplementary file 2 — Supplementary file2 (TIF 4133 KB) [file 299_2024_3213_MOESM2_ESM.tif]

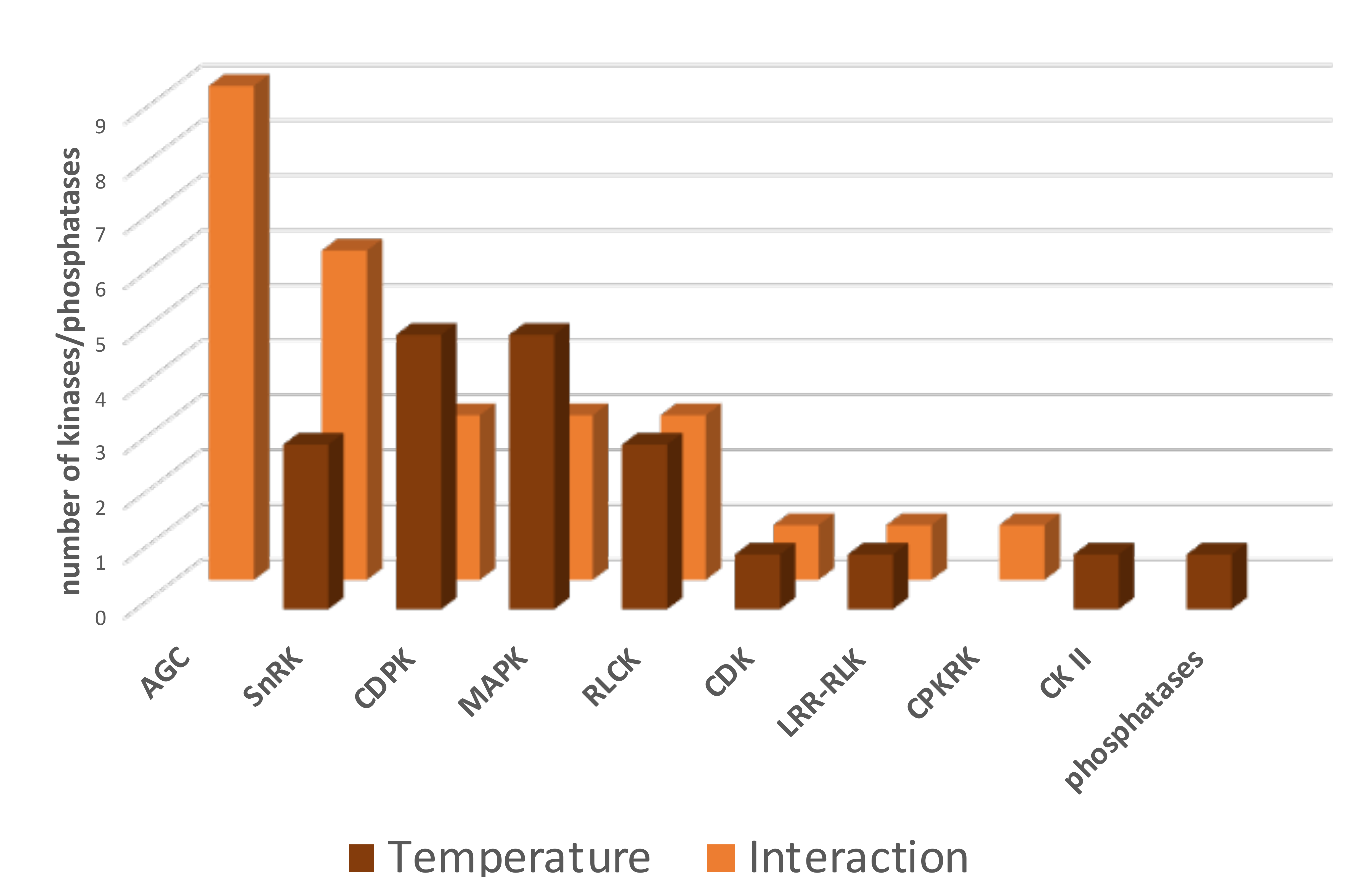

Supplement: Supplementary file 3 — Supplementary file3 (TIF 2551 KB) [file 299_2024_3213_MOESM3_ESM.tif]

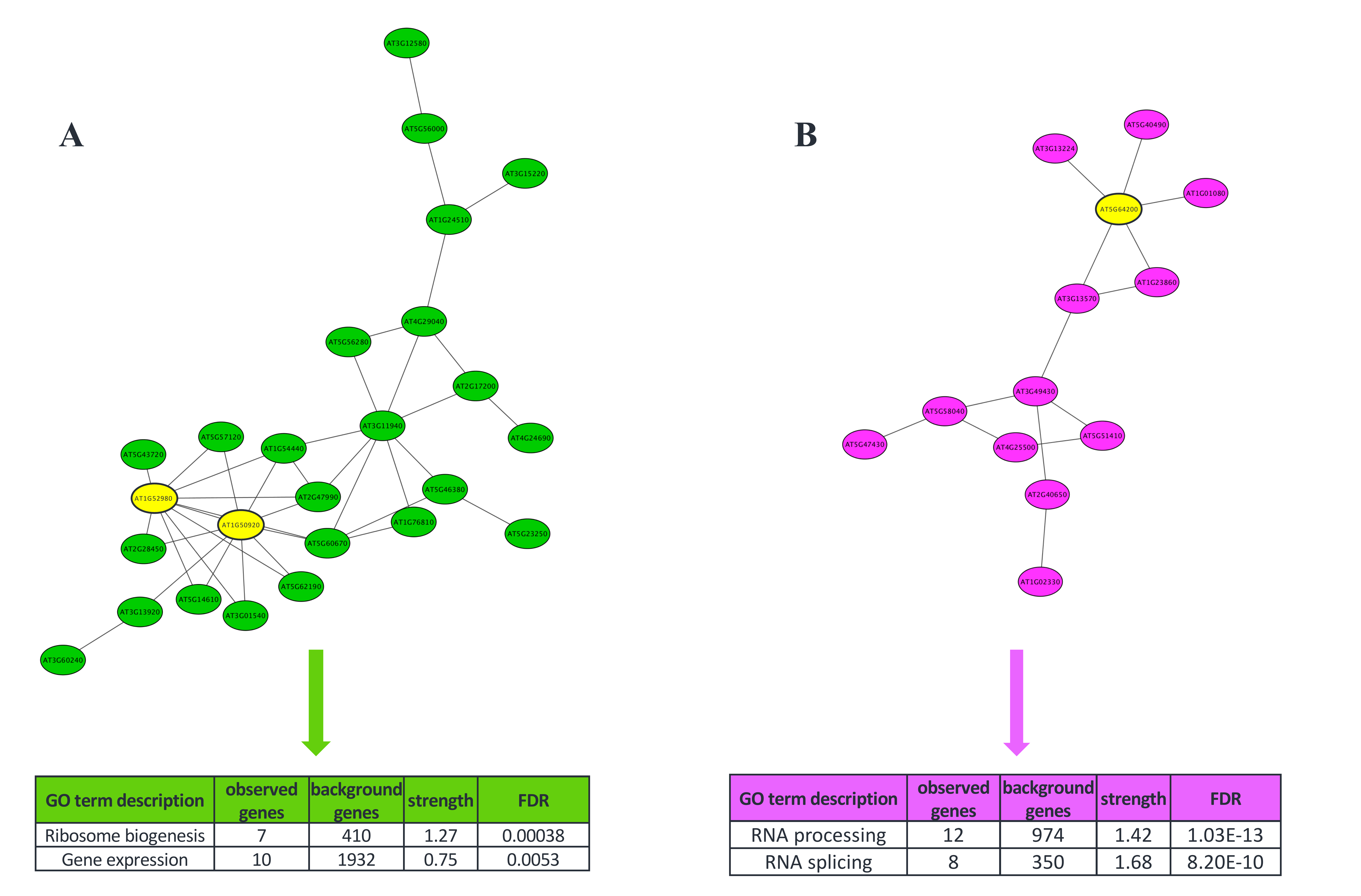

Supplement: Supplementary file 4 — Supplementary file4 (TIF 2163 KB) [file 299_2024_3213_MOESM4_ESM.tif]
